# Machine learning for sustainable organic waste treatment: a critical review

**DOI:** 10.1038/s44296-024-00009-9

**Published:** 2024-04-08

**Authors:** Rohit Gupta, Zahra Hajabdollahi Ouderji, Uzma, Zhibin Yu, William T. Sloan, Siming You

**Affiliations:** 1 UCL Mechanical Engineering, University College London, London, WC1E 7JE UK; 2 Wellcome/EPSRC Centre for Interventional and Surgical Sciences, University College London, London, W1W 7TS UK; 3 James Watt School of Engineering, University of Glasgow, Glasgow, G12 8QQ UK

**Keywords:** Chemical engineering, Environmental sciences

## Abstract

Data-driven modeling is being increasingly applied in designing and optimizing organic waste management toward greater resource circularity. This study investigates a spectrum of data-driven modeling techniques for organic treatment, encompassing neural networks, support vector machines, decision trees, random forests, Gaussian process regression, and *k*-nearest neighbors. The application of these techniques is explored in terms of their capacity for optimizing complex processes. Additionally, the study delves into physics-informed neural networks, highlighting the significance of integrating domain knowledge for improved model consistency. Comparative analyses are carried out to provide insights into the strengths and weaknesses of each technique, aiding practitioners in selecting appropriate models for diverse applications. Transfer learning and specialized neural network variants are also discussed, offering avenues for enhancing predictive capabilities. This work contributes valuable insights to the field of data-driven modeling, emphasizing the importance of understanding the nuances of each technique for informed decision-making in various organic waste treatment scenarios.

## Introduction

With the advent of rapid industrialization, greenhouse gas (GHG) emissions across the globe have increased by 145% over the past five decades^1^, while the generation of organic waste such as food waste and sewage sludge is skyrocketing, reaching 1.75 billion tonnes/year and accounting for one-quarter of overall GHG emissions^2,3^. This poses an alarming concern requiring immediate technological know-how and effective socioeconomic policymaking toward efficient and low-carbon organic waste management. Various types of thermochemical and biochemical waste treatment technologies developed over the past decades have shown promising potential for organic waste treatment, reducing carbon footprint, and contributing to resource recovery towards a circular economy^4^. Some of the popular technologies are gasification, pyrolysis, hydrothermal treatment, anaerobic digestion (AD), composting, and dark fermentation.

Thermochemical technologies (e.g., gasification, pyrolysis, and hydrothermal treatment) utilize thermal energy at high temperatures (and sometimes high pressure) to decompose the organic matter present in the feedstock, converting it to value-added products. In contrast, biochemical technologies (e.g., AD, composting, and dark fermentation) rely on different types of bacteria to decompose the organic matter^4^. The technological selection depends on the scale and operation of systems, type and composition of feedstock, and types of products desired. The efficiency, stability, and carbon footprint of these waste treatment technologies depend on the combination of feedstock and technology, system configuration, and process conditions, which renders optimal process control challenging.

A wide variety of coarse-grained or fine-grained kinetic and thermofluidic models have been explored to simulate and control the behavior of these processes. The coarse-grained kinetic models are often based on ordinary differential equations, where the spatial variations of physical quantities (e.g., temperature, pressure, and concentration) within the reactor are constant^5^. Several examples of this class of model are anaerobic digestion model 1 (ADM1), acidogenesis-methanogenesis model, Gompertz model, etc^6^. Fine-grained models consider the variations of physical quantities within the entire spatial domain, thus require solving partial differential equations to simulate the process behavior. A classic example of this type of model is the computational fluid dynamic (CFD) model of thermo/bio-chemical reactors which simulates both thermofluidic and kinetic phenomena. Despite the potential to achieve high prediction accuracy, these models are computationally intensive, which makes them less feasible for design optimization and real-time process control purposes.

The shortcomings of the kinetic and thermofluidic models can be addressed by data-driven models based on machine learning (ML). Typical benefits that an ML-based model can offer are (1) shortening model computation time, (2) avoiding recalibration, (3) opportunity for embedding physical laws in the data-driven framework, and (4) relatively easy integration within the control system framework^7^. A variety of ML models has been explored by the organic waste treatment research community e.g., neural network (NN), support vector machine (SVM), logistic regression (LR), random forest (RF), eXtreme gradient boosting (XGBOOST), *k*-nearest neighbors (KNN), etc. These models were used to predict the yield and composition of the output products, stability of the process, or environmental footprint^7^.

**Fig. 1:**
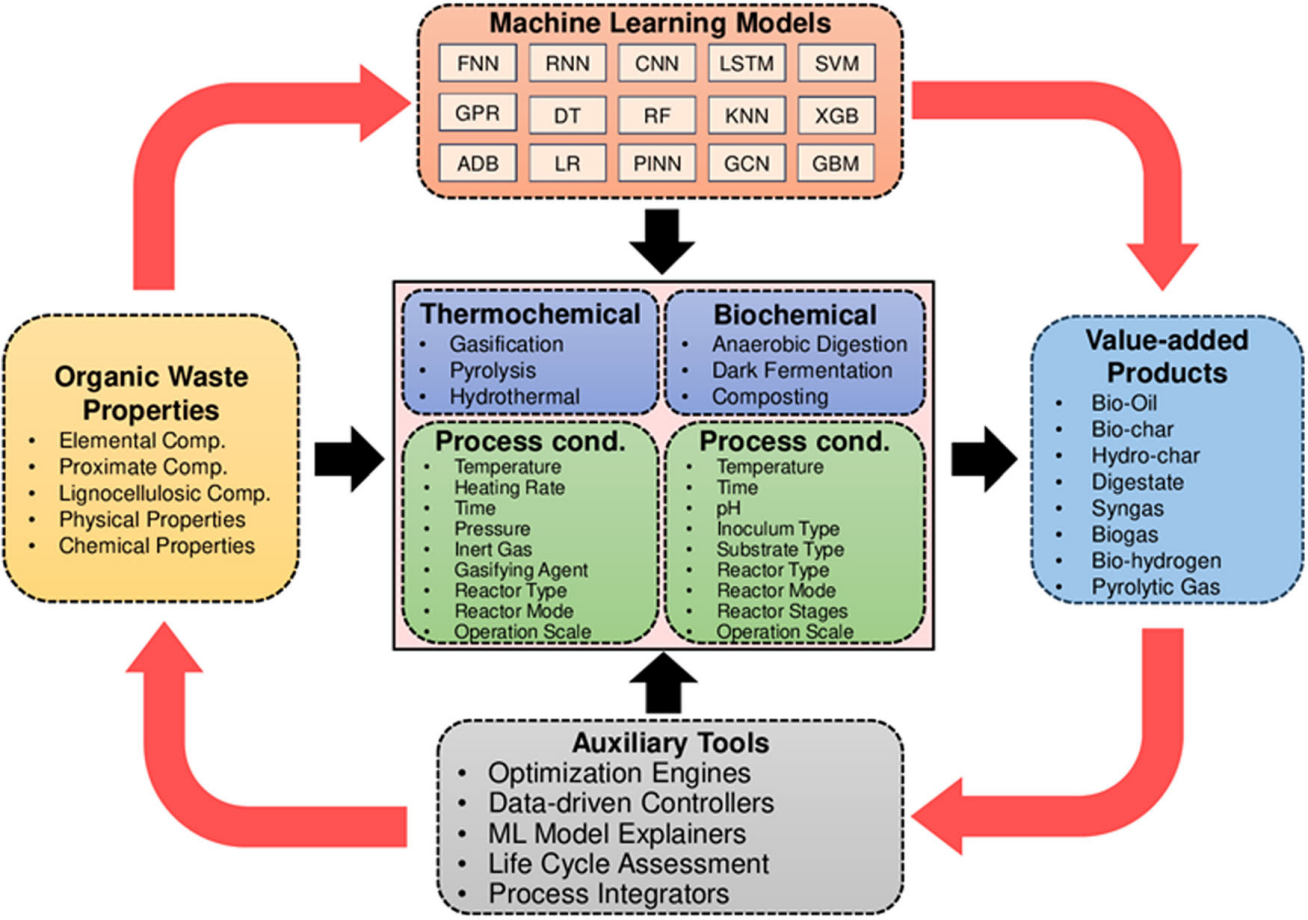
Thematic overview covering organic waste valorization technologies, ML methods, and process optimization tools. Each of the dash-line box lists a range of frequently occurring keywords in the literature.

Despite the rapid development of the data-driven models in organic waste treatment modeling, there is lack of an up-to-date, comprehensive summary of waste treatment technologies and the associated usage of ML-based data-driven modeling. This work aims to critically review existing applications of the data-driven models in bioprocessing including process parameter optimization, control system implementation, and *what-if* scenario analysis (see Fig. 1). To facilitate the ML-related discussion, the principles of six major organic waste treatment technologies are briefly introduced firstly. This comprehensive study also focuses on highlighting the integration of ML-based modeling with environmental impact assessment models and addressing various challenges in popularizing ML-based biological process modeling, as well as potential mitigation strategies. It offers insights and recommendations for increasing the use of ML-based data-driven modeling for facilitating the application and optimal control of sustainable waste management techniques.

## Organic waste treatment technologies

**Fig. 2:**
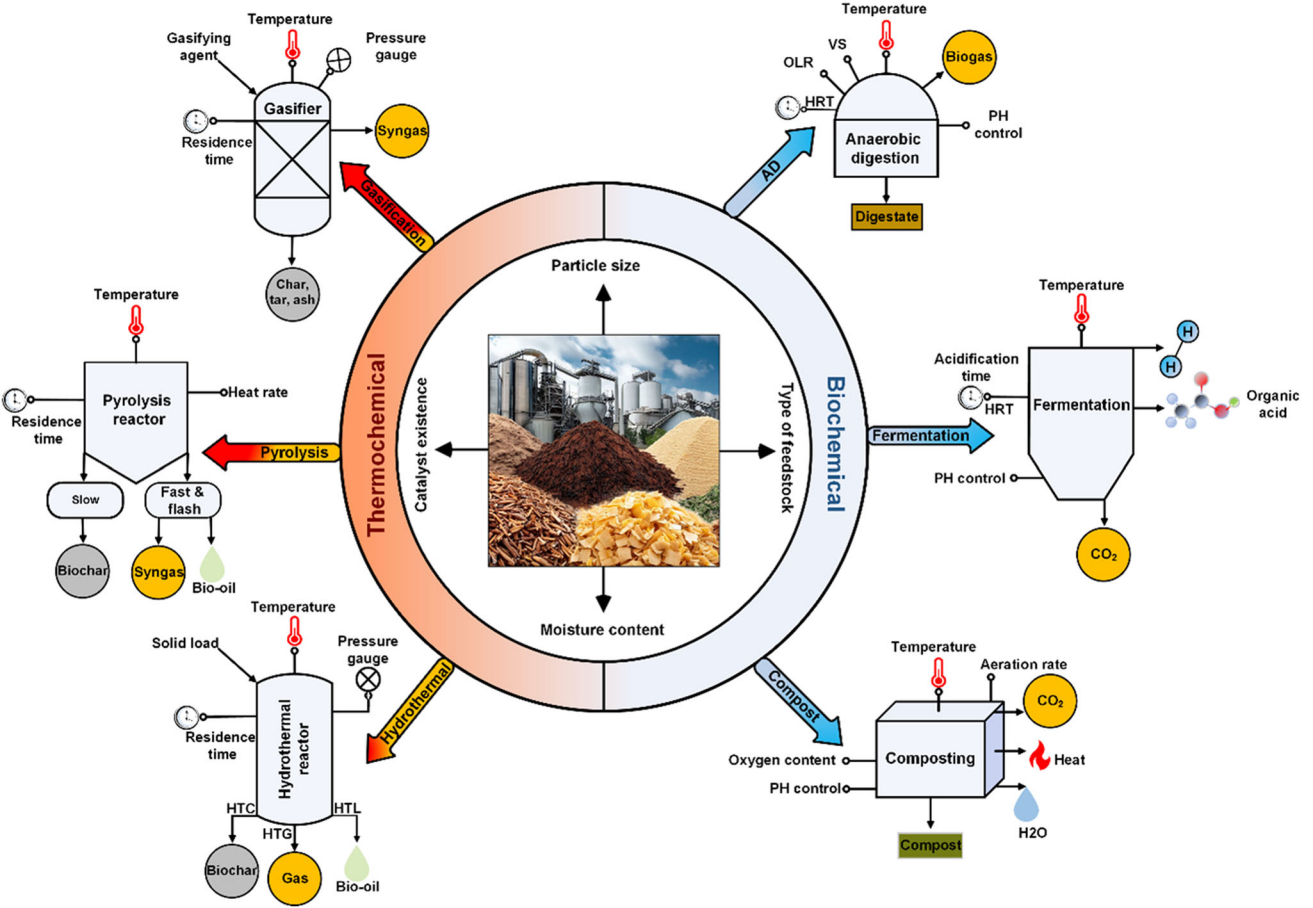
Overview of thermochemical and biochemical organic waste treatment technologies. Each of the technology figure includes examples of input feedstocks, process control parameters, and examples of output product(s).

Popularly used organic waste treatment technologies either use thermal energy or biological microorganisms to break down organic matter within the feedstock. Upon waste decomposition, a three-phase product mixture is often formed consisting of solid, liquid, and gaseous products, whose yield varies based on the selection of technology. For example, gasification promotes the production of syngas, which is a mixture of several value-added gases, while slow pyrolysis promotes the production of biochar or fast pyrolysis predominantly produces bio-oil^4^. Figure 2 shows conceptual schematics of six popular thermochemical and biochemical waste treatment technologies namely gasification, pyrolysis, hydrothermal treatment, AD, composting, and dark fermentation.

### Gasification

Gasification converts organic waste into syngas (a mixture of CO, H_2_, CO_2_, H_2_O, CH_4_, and higher-order hydrocarbons), ash, and biochar under an oxygen-deficient condition of 500–1200 °C^8^. The yield and properties of the end products are regulated by various factors such as properties and size of feedstock, gasifying agent, temperature and pressure within the reactor, reactor design, and the addition of catalysts and sorbents^9^. One of the major challenges associated with the use of gasification is tar formation, which adversely affects the efficiency and commercial viability of this process^10^. Auxiliary processes such as catalytic tar reforming, scrubbing, thermal cracking, and filtering have been proposed to mitigate the issue^11^. Recent research explored the impact of a dielectric barrier discharge reactor on biomass gasification, offering insights into CO_2_ decomposition, CO concentrations, and tar reduction^12^. Concurrently, research was conducted on supercritical water gasification, with an emphasis on greater hydrogen production and less tar formation^13^. In addition, hybrid waste-to-energy systems that combine waste gasification with renewable energy resources and AD were investigated to enhance the viability and effectiveness of the process^14^.

The gasification processes involve a large pool of chemical reactions and heat and mass transfer formulations. Multi-phase CFD simulations have been developed to understand the output product variations as a function of the input feedstock and scale of operation^15^. However, one major limitation of the simulations is their incapability to account for all influential factors and process conditions, thus constraining their application for optimal process design and control. In contrast, ML-based gasification modeling has been carried out to predict the influences of a comprehensive range of factors (e.g., reactor type, catalyst usage, gasifying agent and temperature, feedstock types, etc.) towards the yields of syngas components and biochar^16^.

### Pyrolysis

Under an oxygen-free condition of typically 200–500 °C, pyrolysis converts organic waste into a mixture of biochar, bio-oil, and gaseous products^17^. This process is featured by lower CO_x_, SO_x_, and NO_x_, emissions than the gasification process, and is desired if biochar and bio-oil are target products^18^. Building upon the understanding that the efficacy of pyrolysis is intricately linked with feedstock characteristics and process conditions (e.g., temperature, heating rate, residence time, etc.), recent studies delve into advancing the technology by altering the adaption of heating and reaction mechanisms^19^. One such example is the proposal of microwave-assisted pyrolysis (MAP), which heats biomass quickly and evenly using microwave radiation. Co-pyrolysis of organic waste, a novel method has the potential to handle multiple waste streams and improve solid/gaseous product yield^20^. Catalytic pyrolysis has been widely studied by changing the reaction pathways using catalysts and has the potential to improve the process stability and selectivity of the final product^21^. Although traditional models based on the analysis of mass transfer, heat transfer, and chemical kinetics have proven important for understanding biomass pyrolysis, their application for controlling and accurately predicting the process is limited due to the inability to fully account for the complex structure of the feedstock and the process, which involves more than a hundred intermediate products^22^. To overcome these challenges, ML-based modelling proves to be a useful tool, providing a data-driven and adaptive method for a more precise and adaptable prediction of pyrolysis processes^23^.

### Hydrothermal treatment

Hydrothermal treatment is suitable for organic waste with a high moisture content and is not contingent upon a drying pretreatment stage, which is often the case for gasification and pyrolysis. Compared to gasification, hydrothermal treatment is carried out at a lower temperature (250–374 °C), high heating rates, and pressure in the range of 4–22 MPa^24^. Influential factors of the process are temperature, pressure, residence time, type of catalyst used, and feedstock composition^25^. There are three types of hydrothermal waste valorization technologies: hydrothermal gasification (HTG), hydrothermal liquefaction (HTL), and hydrothermal carbonization (HTC), that differ by the choice of output product. The HTG process uses a supercritical condition, in which the waste reacts with water at high temperature and pressure to produce a mixture of gaseous products consisting of CH_4_, H_2_, CO_2_, CO, and C_2_-C_4_^26^. The major drawback of the process is the usage of large quantities of heat and water. In the HTL process, both subcritical and supercritical conditions are used for producing bio-oil. Subcritical liquefaction occurs around the critical point of the liquid (e.g., 374 °C for water), while supercritical liquefaction uses temperatures and pressures above the critical point. Being a fluid-dominant process, the HTL process often suffers from equipment corrosion and catalyst deactivation^27^. In addition, hydrochar is the outcome of HTC that is formed from a slurry in water under high pressure and low temperature. The produced hydrochar can be used as a catalyst, adsorbent for pollutants, material for carbon sequestration, soil amendment, or as an energy carrier^28^.

### Anaerobic digestion

The AD process is conducted under oxygen-free conditions and uses bacterial microorganisms to yield biogas comprising mainly CH_4_ and CO_2_. The byproduct, digestate, includes various nutrients like nitrogen, phosphorus, and potassium, making it suitable for use as a fertilizer or soil conditioner for enhancing crop productivity and promoting carbon sequestration^29^. The AD process has been vital for curbing pollution from organic waste in agriculture and industry. It can handle a diverse range of feedstocks ranging from agricultural waste, organic matter-rich industrial waste, animal waste, sewage sludge, and woody waste^30,31^. The second product, biogas, finds applications in heating, power generation, and as a transport fuel after upgrading.

The complexities of input factors, such as feedstock types and compositions, temperature, pH, hydraulic retention time (HRT), and OLR, contribute to the complex and non-linear impact of these parameters on AD output^32,33^. This level of complexity makes optimizing the treatment process difficult, emphasizing the importance of accurate and effective approaches for predicting their influence on the AD system. ML can be applied in AD to predict the outputs, optimize processes, and effectively control AD performance, addressing operational challenges such as process instability and disruptions in microbial activities, which can lead to reduced methane generation^34,35^.

### Dark fermentation

Fermentation, a process converting organic substrates into valuable products like biohydrogen and bioethanol, encompasses photo- and dark fermentation occurring in light and dark settings, respectively. Dark fermentation, particularly applied for treating organic waste, offers advantages such as moderate reaction conditions and higher production rates compared to photo fermentation^36^. This sustainable technology, focused on biohydrogen production, faces challenges due to its low yields, limiting industrial applications^37^. In the initial step, bacterial hydrolysis breaks down substrates into smaller molecules, which are further fermented into organic acids during acidogenesis^38^. Organic substrates abundant in carbohydrates, like starch and cellulose, prove feasible for dark fermentation-based biohydrogen production^39^. However, methanogen proliferation can limit biohydrogen production from certain feedstocks, affecting purity^40^. Temperature variations (mesophilic, thermophilic, hyper-thermophilic) significantly impact bacteria growth rates and substrate conversion efficiency^41^.

ML models can be employed to achieve effective process control and production, addressing the challenges faced by dark fermentation and unlocking greater efficiency and yields^42^. Various influential process parameters associated with dark fermentation should be considered by ML models including feedstock types, temperature, substrate ratios, acidification time, chemical oxygen demand (COD), substrate pH, HRT, and reactor types^43^. The consideration of a wide range of numerical (e.g., HRT, temperature, etc.) and categorical (e.g., feedstock type, reactor type, operating mode, etc.) variables may effectively overcome challenges posed by mechanistic models for predicting dark fermentation-derived biohydrogen yield^44^.

### Composting

Composting, a process of decomposing organic waste into a stable and nutrient-rich product for plant growth, relies on naturally occurring soil microorganisms and requires oxygen^45^. This waste diversion method significantly contributes to waste management by preventing landfill disposal^46^. The composting process can be divided into mesophilic, thermophilic, and maturation stages based on temperature, which is a key factor influencing microorganism activity and organic matter decomposition^47^. Various composting systems, such as static, rotating, windrow, and silo, influence the composting maturity period and the overall process efficiency^48^. Specialized equipment like silos, tunnels, and aerated containers aids in the composting process^49^. The composting process generates GHGs and volatile compounds, requiring emissions management strategies such as the development of biofilters, which can be facilitated by accurate ML modeling of the GHG emission of composting^50^.

To address the challenge of reducing GHG emissions in the composting process, ML techniques are developed^51^. These techniques not only enable the accurate prediction of GHG output but also extend their capabilities to forecasting composting processes and their impacts, especially upon soil application. Recent studies have focused on using ML to predict composting stability and performance, emphasizing its potential to significantly enhance precision in anticipating outcomes and optimizing the entire composting process^52^. Influential factors crucial for ML modeling in composting encompass feedstock types, temperature, pH, carbon-to-nitrogen (C/N) ratio, cation exchange capacity, seed germination index^53^, substrate particle size, moisture content, and composting methods^54^.

## Data-driven modeling techniques

**Fig. 3:**
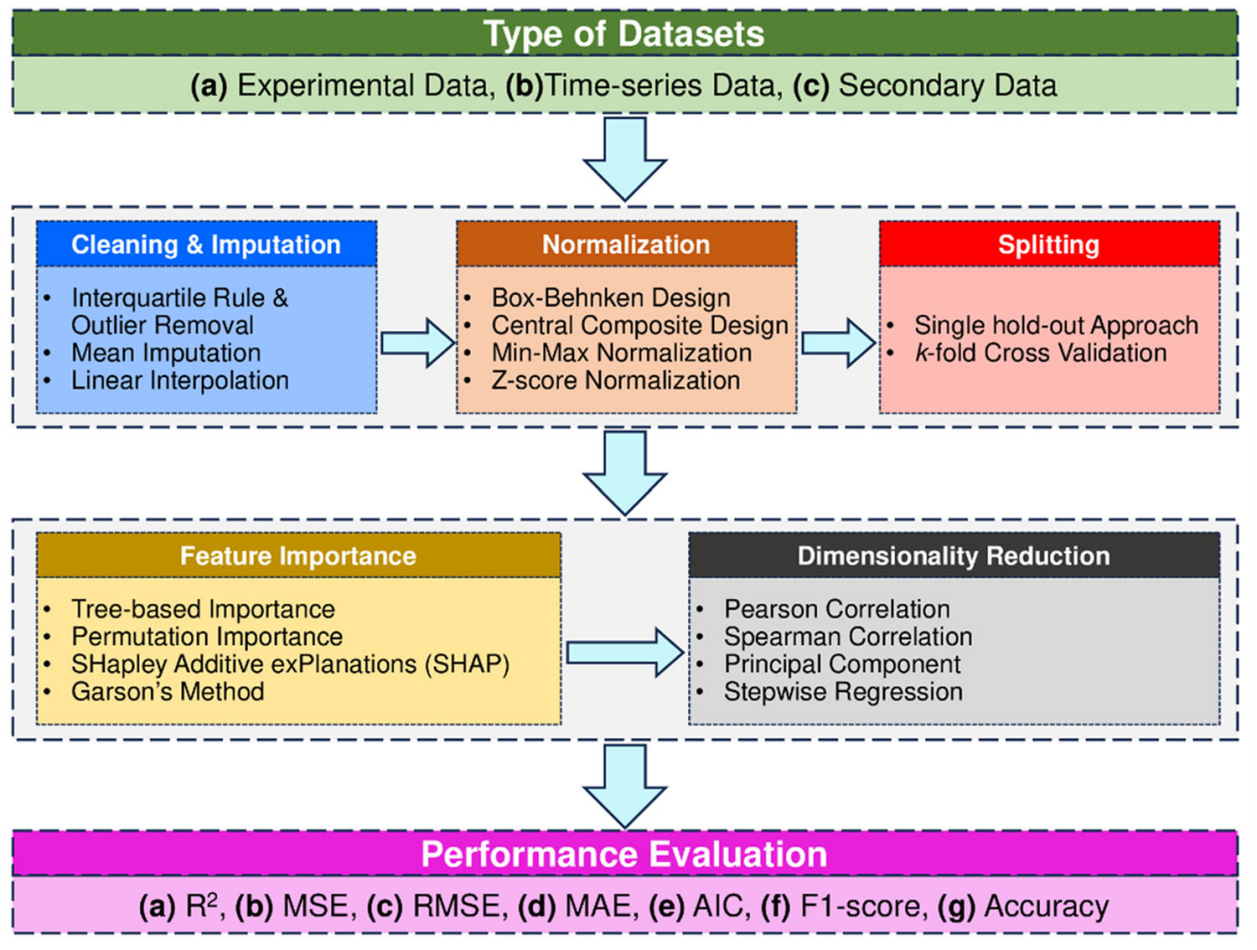
Sequential stages involved in data-driven model development ranging from dataset construction to performance evaluation. The stages include creation of a specific type of dataset, data pre-processing and normalization, model complexity reduction, interpretability analysis, and performance evaluation.

ML-based data-driven models serve as effective tools to predict the process dynamics of the thermochemical and biochemical organic waste treatment technologies described in Section “Organic waste treatment technologies”, towards accurate process design, control, and optimization. Development of these data-driven models require a series of stages starting from data collection, statistical pre-processing, normalization, dataset splitting, dimensionality reduction, feature importance analysis, to model performance evaluation. A comprehensive layout of these stages and their essential attributes relevant to ML-based organic waste treatment is shown in Fig. 3.

**Fig. 4:**
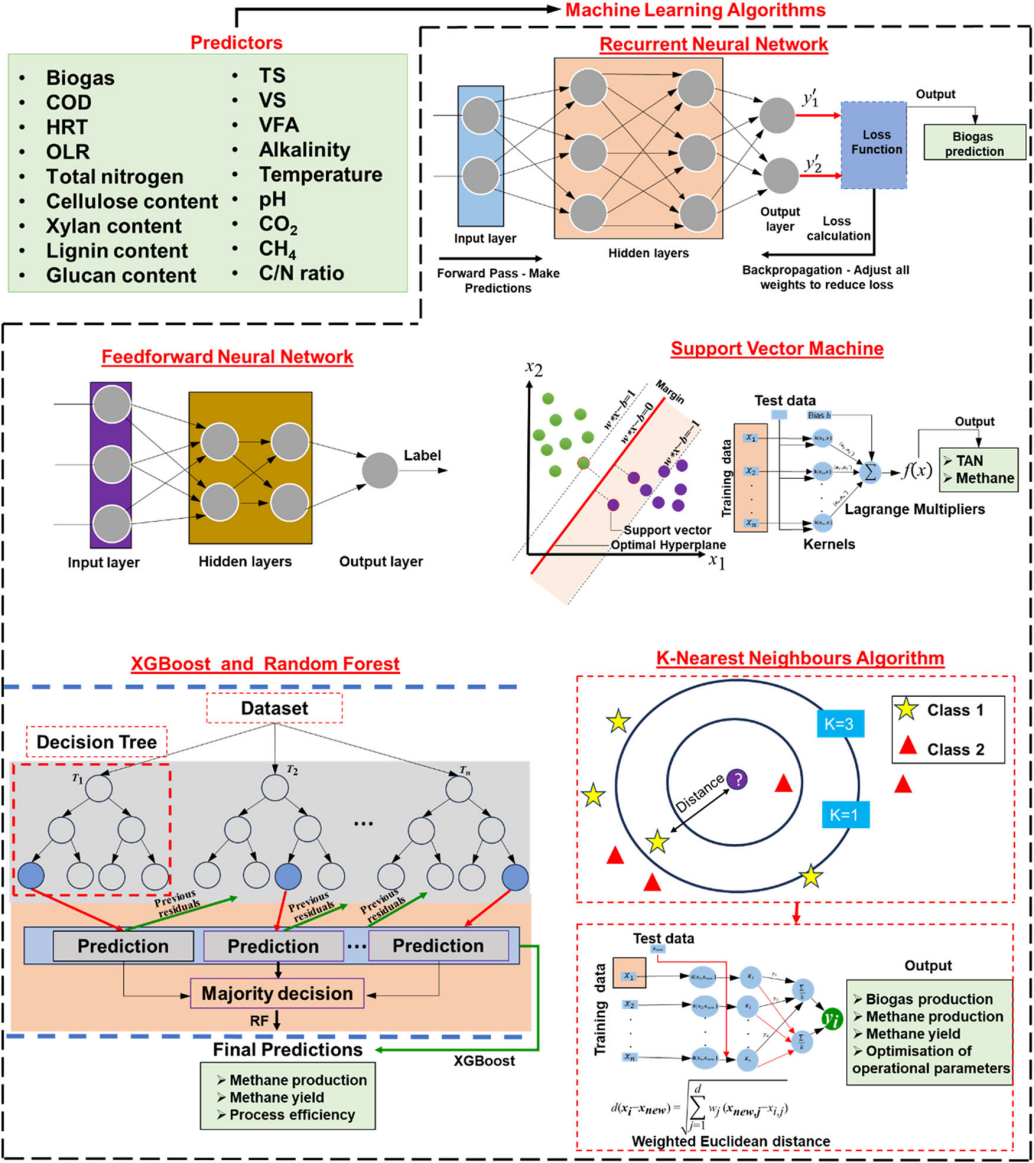
Frequently used ML-based data-driven models for thermochemical and biochemical organic waste treatment processes. It includes examples of predictor variables used to construct data-driven models for organic waste valorization technologies, architectures of RNN, FNN, SVM, XGBOOST, RF, and KNN algorithms, and examples of target variables.

The general ML model development pipeline shown in Fig. 3 can be affected by the selection of data-driven model. Different ML models feature different forecasting abilities based on the input dataset, varied degrees of generalizability and robustness, executing and training time, and the ability to embed physicochemical phenomena in it. A total of eight different types of data-driven models are reviewed here (see Fig. 4), that have been frequently used for modeling organic waste treatment technologies: NN, physics-informed neural networks (PINN), SVM, decision tree (DT), ensembled DT, Generalizable Linear Model with ElasticNet regularization (GLMNET), Gaussian process regression (GPR) and KNN^7,16,52,55^. When deployed for modeling organic waste treatment technologies, these models predict either static or time-series data of process efficiency, yields of products (e.g., biogas, syngas, bio-crude, biochar, hydrochar, etc.), or amount of nutrients recovered from organic waste (e.g., nitrogen, phosphorus, potassium, etc.). These predicted variables are correlated with a variety of input (or predictor) variables, based on the selected thermochemical or biochemical treatment technology. Table 1 provides a generalized overview of the predictor and target variables related to ML-based modeling of organic waste treatment^56^.

**Table 1:** Predictor and target variables for data-driven modeling of six different organic waste treatment processes

Technique	Predicted Variables	Predictor Variables	Usage
Gasification	Syngas yield and composition	Temperature, equivalence ratio, feedstock ultimate composition (C/H/O/N)	Frequent
		Steam-to-feedstock ratio, calcium oxide-to feedstock ratio, feedstock ash and moisture contents	Moderate
		Time, organic loading rate, particle diameter, fuel and air flow rates, pressure, volatile matter and fixed carbon in feedstock, total solids, catalyst usage	Rare
Pyrolysis	Biochar yield (slow pyrolysis) or bio-oil yield (fast pyrolysis)	Temperature, residence time	Frequent
		Particle diameter, heating rate, inert gas flow rate	Moderate
		Organic loading rate, pressure, feedstock ultimate composition (C/H/O/N), feedstock proximate composition (fixed carbon, ash, volatile matter), feedstock structural composition (lignin, cellulose, hemicellulose), catalyst usage	Rare
Hydrothermal Treatment	Hydrochar (HTC) or bio-crude (HTL) or syngas (HTG) yields	Temperature, residence time	Frequent
		Feedstock-to-water ratio, feedstock ultimate composition (C/H/O/N), ash content	Moderate
		Organic loading rate, heating rate, pressure, feedstock proximate contents (fixed carbon, volatile matter, moisture content), total solids, feedstock structural composition (lignin, cellulose, hemicellulose), other biochemical contents (lipid, protein, carbohydrate), catalyst usage	Rare
Anaerobic Digestion	Biogas or methane yields	Temperature, time, pH, feedstock quantity	Frequent
		Total solid, volatile solid, lignin content	Moderate
		Organic loading rate, chemical oxygen demand, alkalinity, ammoniacal nitrogen, C-to-N ratio, feedstock structural and biochemical compositions, acid detergent fiber, catalyst usage, feedstock pretreatment effect	Rare
Dark Fermentation	Biohydrogen yield	Time, temperature, substrate concentration, pH	Frequent
		Chemical oxygen demand, volatile fatty acid, inoculum (type, age, size)	Moderate
		Reactor/feed type, volatile solids, organic loading rate, reactor volume, alkalinity, oxidation reduction potential, catalyst usage, glucose-xylose ratio, glucose concentration, lactate, acetate, propionate, butyrate	Rare
Composting	N and P contents	C-to-N ratio, pH, electrical conductivity, temperature, feedstock quantity	Frequent
		Time, moisture content, enzyme type, dry solids, ammonium-to-nitrate ion ratio	Moderate

### Neural network

An NN is inspired by the structure and functioning of the human brain, comprising the following subunits: neurons, hidden layer, input layer, and output layer^57^. Based on a preselected architecture, the NN applies a series of activation functions and assigns weights and biases, to map the input (feature) space to the output variables. Choice of the activation function is an essential aspect of developing an NN, where some of the frequently used choices are sigmoid, hyperbolic tangent, rectified linear unit (ReLU), and leaky ReLU. Fitting the NN to a specific dataset requires adjusting the weights and bias factors, which is done through a process called backpropagation. The process uses gradient descent to minimize the loss function computed as a root mean squared error (RMSE) between the true dataset and NN predictions. Based on the area of applications, complexity, and type of dataset (e.g., high or low dimensional, static or dynamic dataset, image-based spatial datasets, etc.), a wide variety of NNs have been explored such as feedforward NN (FNN), recurrent NN (RNN) with long short-term memory (LSTM) networks, convolutional NN (CNN), transformers, etc. The complexity (i.e., type of architecture, number of tuneable parameters, etc.), training, and execution times of each type of NN differ. Nevertheless, it is important to note that having an NN with complicated architecture might lead to model overfitting, thus reducing the generalizability of the model.

FNN is one of the most classical types of NN, generally containing several neurons per layer and being several layers deep. The non-linear activation functions of the neurons enable FNNs to gain complex data reproducing capability. This type of NN is a powerful tool for both regression and classification tasks^58^. Since data-driven models required for bioprocess modeling might require both regression (e.g., the influence of control parameter alteration on system efficiency) or classification (e.g., process failure prediction, reactor anomaly identification and alarm generation), FNNs has been frequently used in data-driven bioprocess modeling^7^. Nevertheless, FNNs are susceptible to the curse of dimensionality, require normalization and data preprocessing, and are not suitable for capturing time-series data trends. Time-series data can often be encountered about the temporal yield of gaseous products from organic waste treatment.

The time-series data processing inability of FNNs has been addressed by RNNs, which are suitable for processing audio signals, sensor readings, financial data, epidemiology data, etc. RNNs contain feedback loops that allow information to persist across time steps, making them adept at capturing temporal dependencies within the data. Despite the powerful capability of RNNs, they often face vanishing or exploding gradients during backpropagation-based training, which have been carefully addressed by advanced techniques such as a long-short-term memory (LSTM) network^59^. LSTM networks excel in managing long-range dependencies and are well-suited for various sequential data processing tasks. At the core of an LSTM networks is the LSTM cell, which processes input data sequentially and maintains a hidden state that captures relevant information from previous time steps. The cell consists of three key gates such as input, forget, and output gates.

The curse of dimensionality faced by the FNNs can be addressed by the CNNs, a specialized deep learning method frequently used for processing image or grid-like data. They consist of a convolutional layer for learning local patterns from high-resolution data, a pooling layer for reducing spatial dimensions, and fully connected layers (like FNNs) for learning non-linear patterns^60^. The convolution operation, which is the backbone of CNNs, involves sliding a filter over the input and computing dot product to generate feature maps that foster automatic hierarchical feature learning. The CNN architecture enables weight sharing across the network, which greatly reduces the number of parameters and promotes translation-invariant features. Like FNNs, training CNNs involves backpropagation and gradient descent to minimize the loss function. CNNs have evolved with variants like VGG, Inception, and ResNet to tackle specific challenges like overfitting and vanishing gradients. Another version of CNN, named one-dimensional CNN (1D-CNN) has demonstrated high effectiveness in dealing with sequence-based time-series data.

In addition to FNN, RNN-LSTM, and CNN, transformer networks have evolved as an efficient data-driven model for mimicking sequential data with complex trends. Specifically, in the realm of predicting bioprocessing outcomes for organic waste, transformer networks demonstrate efficacy in capturing intricate temporal dependencies and patterns within the data. This makes them well-suited for tasks such as predicting gas yield variations over time or anticipating efficiency fluctuations in organic waste treatment processes. The transformer architecture excels in handling sequential data, making it applicable to time-series datasets commonly encountered in bioprocess modeling of organic waste treatment.

Transformers leverage self-attention mechanisms to capture dependencies between different positions in a sequence, enabling them to excel at tasks involving long sequences and parallel processing. Combining transformers with transfer learning techniques helps to leverage knowledge gained from one task to improve performance on a different but closely related task. In the context of bioprocess modeling, transfer learning could be explored to enhance the predictive capabilities of the models by pretraining them on a large dataset, such as general time series data of bioreactors, before fine-tuning them on the specific task of optimization.

### Physics-informed neural network

Physics-informed neural network (PINN) is a class of deep NNs, that informs the power of a data-driven NN with governing conservation equations of physical principles (e.g., mass, energy, momentum, or species)^61^. By construction, any solution predicted by a PINNs will satisfy the governing equation, thus producing a physically realistic solution in all instances. PINNs can incorporate algebraic, ordinary differential, or even partial differential equations to enforce the physics inside the NN. The major difference between PINNs and an ordinary FNNs is the construction of the loss of term which is trained using stochastic gradient descent. An ordinary FNN minimizes the RMSE between the input dataset and FNNs predictions, while a PINNs adds additional loss terms: different types of loss terms can be constructed to penalize the PINNs such as losses for governing equations, boundary conditions, initial conditions, etc. Construction of these equation losses requires utilizing the power of automatic differentiation, a tool that makes PINNs a grid-free method for solving differential equations. Different types of losses (e.g., RMSE loss, governing equation loss, and initial and boundary condition losses) included in the overall loss function must be weighted properly to ensure the accurate behavior of the PINNs^62^. Once trained, PINNs can predict spatiotemporal data (e.g., pressure, velocity, concentration, temperature, density, etc.) in an extremely rapid manner, which is often a challenge for grid-based methods for solving differential equations. PINNs find a promising application in enhancing organic waste treatment technologies. Their capability to incorporate governing conservation equations equips them to model and optimize various processes within waste treatment. In waste treatment, PINNs, with their physics-informed nature, provide accurate predictions, offering valuable insight for parameter optimization and efficiency improvement. The rapid predictions of spatiotemporal data by PINNs address challenges posed by grid-based methods in handling differential equations within the dynamic systems inherent in organic waste treatment^63^. Furthermore, the integration potential of PINNs with other data-driven methods positions them as attractive candidates for control-oriented operations, fault diagnosis, and decision-making within the realm of organic waste treatment technology. The versatility of PINNs, coupled with their adept handling of specific challenges associated with organic waste treatment, establishes them as a noteworthy asset for advancing and optimizing processes in this domain.

### Support vector machines

SVM is a robust supervised learning algorithm widely utilized for classification and regression tasks^7^. The key objective of SVM is to identify the optimal hyperplane that effectively separates data into different classes while maximizing the margin between them. This robustness and generalization performance is attributed to the kernel trick, allowing SVMs to handle both linear and nonlinear classification problems, even in scenarios with limited samples^64^. In terms of performance, SVMs are renowned for their ability to handle high-dimensional data and complex decision boundaries. The kernel trick enables them to address nonlinear relationships between features, providing an advantage over linear models. SVMs exhibit robust generalization, making them suitable for various ML tasks.

Comparing SVM with other models like DT, KNN, LR, and NN reveals the apparent effectiveness of SVM, especially in scenarios with high-dimensional data and intricate decision boundaries. High-dimensional data, often encountered in organic waste treatment scenarios, refers to datasets with numerous variables, such as waste composition, environmental conditions, and treatment processes. In these scenarios, where relationships can be intricate, SVM demonstrate proficiency by effectively handling the complexity of the data and decision boundaries, making them a noteworthy choice for modeling and optimizing organic waste treatment processes. The one-vs-all (OvA) or one-vs-one (OvO) techniques extend SVMs to handle multiclass problems, showcasing their versatility in tackling diverse classification challenges^52^. As for tuneable parameters, SVMs offer several key parameters that can be adjusted for optimal performance. These include the choice of kernel (linear, polynomial, radial basis function, etc.), regularization parameter, and kernel-specific parameters. Proper tuning of these parameters is essential for achieving the best results in different scenarios.

### Decision trees

DT is a supervised ML algorithm used for both classification and regression tasks^34^. It constructs a tree-like structure where each internal node represents a decision or test on a feature, and each leaf node signifies a predicted class (for classification) or a numeric value (for regression). Key components of DT include the root node, internal node, leaf nodes, branches, and attributes.

Examples of open-source DT algorithms include ID3, C4.5, and Classification And Regression Trees (CART), which vary by their dataset splitting and pruning methods. Commonly used splitting methods include Gini impurity (for classification), entropy (for classification), and mean squared error (MSE, for Regression). The splitting rule at each node aims to minimize impurity or MSE by selecting the attribute that achieves this goal. Pruning is employed to prevent overfitting by removing branches that do not significantly improve predictive accuracy^7^. The feature selection methods (e.g., Gini impurity) adds an incentive by mapping the strength of correlation across the predictor and target variable, making DTs explainable ML models.

In DTs applied for classification tasks, a new data point follows the decision rules to reach a leaf node, determining the predicted class. In regression tasks, the predicted value is the mean or median of the target values in the leaf node. However, DTs are severely prone to overfitting which sacrifices the model generalizability. To circumvent this, ensembling of multiple DTs have been routinely practiced, a topic that will be discussed in later sections. Nevertheless, DTs are featured by their short training and evaluation times, making them suitable for rapid predictive modeling of organic waste treatment. Of great relevance is applications such as yields prediction of solid/liquid/gaseous products based on a small set of predictor variables (usually <5) or binary fault identification associated with bioreactors^65^.

### Ensembled decision trees

The ensemble learning method mitigates the overfitting limitations (i.e., lack of model generalizability) of individual DT. There are two types of ensemble methods: bagging and boosting. The bagging approach creates multiple trees through a process called bootstrapping, where each tree trains on a random subset of data^16^. A routinely used bagged ensemble method is RF, which introduces feature randomization by considering only a subset of features at each tree split, reducing sensitivity to specific features. When making predictions, RF combines results through majority voting for classification and averaging for regression, resulting in reduced overfitting, improved generalization, stronger predictive power, and robustness to noisy data.

RF demonstrates exceptional performance when dealing with extensive, high-dimensional, noisy, and imbalanced datasets, while effectively mitigating overfitting issues. This is particularly advantageous because DT within the RF ensemble can swiftly learn and process both categorical and numerical data, often without the need for extensive data pre-processing, provided that no assumptions regarding the data’s quality, such as linearity or normality, are mandated. Furthermore, RF distinguishes itself by providing more detailed insights into the significance of input variables compared to alternative methods such as linear regression, SVM, DT, and NNs. Additionally, RF boasts a robust ability to handle missing data, making it a versatile and reliable choice for various data analysis tasks. RF has demonstrated effectiveness across a spectrum of applications, underscoring its capability to model and forecast in scenarios with noisy datasets. In the field of organic waste processing, RF has shown success in optimizing composting processes, accurately predicting the efficiency and decomposition rates despite variations in factors such as temperature, moisture content, and organic waste composition^7^.

Boosting is another ensemble method that focuses on improving the accuracy of a model by combining multiple weak learners (usually individual DT) sequentially. Gradient Boosting Machines (GBMs), including XGBOOST and LightGBM, are examples of boosting ensemble methods^66^. GBMs are known for their ability to handle complex relationships and have been successfully applied in various domains, including regression, classification, and ranking tasks. They offer high predictive accuracy but may require careful parameter tuning. Key parameters for GBM include the number of trees, learning rate, tree depth, minimum samples split, minimum samples leaf, subsample, feature fraction, and regularization parameters. This tuning is crucial for preventing overfitting, and GBMs are often more effective at mitigating overfitting challenges compared to RF. GBMs sequentially build DTs, where each tree corrects the errors of the previous one by assigning higher weights to data points that were previously misclassified or had larger errors.

Among the implementations of GBMs, two popular libraries are XGBOOST and LightGBM. XGBOOST is widely recognized for its speed and efficiency, thanks to features such as tree pruning and parallel processing. It offers L1 (Lasso) and L2 (Ridge) regularization options to prevent overfitting and can handle missing data, making it versatile for both regression and classification tasks^65^. On the other hand, LightGBM is specifically designed for large-scale and distributed settings, excelling in terms of speed and memory efficiency. It is particularly suitable for scenarios with large datasets, making it a potential asset for applications in organic waste treatment, where extensive data on waste composition, environmental conditions, and treatment processes may need to be efficiently processed. Additionally, LightGBM includes built-in support for handling categorical features and simplifying pre-processing, further enhancing its applicability in the field of organic waste treatment.

### Generalizable linear models

Generalizable linear models (GLM) are primarily used for binary classification tasks. When extending to multiclass classification, a variant known as multinomial GLM or Softmax regression is commonly employed. The GLMNET algorithm introduces regularization, combining L1 (Lasso) and L2 (Ridge) techniques to enhance model performance^65^. In multiclass classification applications, where there are more than two classes, Softmax regression, an extension of binary GLM, proves effective in assigning input vectors to multiple classes. GLMNET incorporates L1 and L2 regularization terms in the loss function to counter overfitting and promote feature selection. The optimization process can be carried out using various algorithms, such as coordinate descent and gradient descent. The regularization terms discourage overfitting by penalizing large parameter values and facilitating some parameters to be precisely zero, thereby supporting feature selection. The hyperparameter *λ* controls the strength of regularization, while *α* determines the type of regularization (L1 vs. L2). In practical applications, libraries like GLMNET offer efficient implementations of this algorithm for multiclass Generalized Linear Model with ElasticNet regularization with user-friendly interfaces. These libraries manage the optimization and regularization aspects, enabling users to concentrate on model selection and hyperparameter tuning. The added incentive of better control of regularization is essential for the organic waste treatment process modeling due to associated big datasets comprising of time-dependent process parameters, genomic data, and categorical data. For these benefits GLMNET has received significant interests in organic waste treatment modeling^35,67^.

### Gaussian process regression

GPR is a potent non-parametric Bayesian technique utilized for regression tasks, differing from conventional methods by modeling the underlying function as a distribution over functions, a valuable approach for addressing complex and non-linear data relationships^34^. Uncertainty in GPR is represented by modeling the output for each input point as a Gaussian distribution, allowing the provision of predictions along with associated uncertainty estimates. The choice of the kernel function is pivotal in GPR, determining the similarity between input points and shaping the predicted functions. Common kernels, such as the Radial Basis Function (RBF) and Matérn kernels, offer diverse ways to capture relationships. Hyperparameters in GPR, like the length scale in the kernel function, significantly influence the model’s behavior, often optimized during training. GPR excels in scenarios with intricate relationships, finding extensive use in accurate regression and prediction tasks. The capacity of GPR to furnish uncertainty estimates is particularly valuable in applications where comprehending prediction confidence is essential, such as in decision-making processes. GPR finds application in optimization tasks and Bayesian optimization, contributing to sequential optimization strategies. For time series prediction, GPR not only provides point predictions but also includes confidence intervals around those predictions, enhancing its applicability. GPR exhibits high flexibility, adapting effectively to diverse data types and relationships. Its ability to quantify uncertainty proves crucial in applications where understanding prediction confidence is paramount, and it performs well even with limited data availability. However, GPR’s computational cost can escalate with larger datasets, necessitating the use of efficient approximations and optimization techniques. The selection of an appropriate kernel function is critical, as GPR’s performance is sensitive to this choice. In the context of organic waste treatment, GPR can be employed to model and predict complex relationships within the treatment process, offering precise estimates and uncertainty quantification, crucial for optimizing waste treatment parameters and decision-making.

### *k*-nearest neighbors

KNN is a versatile ML algorithm utilized for both classification and regression tasks^7^. Its operation revolves around the principle of identifying the *k* nearest neighbors in the feature space to make predictions based on their characteristics. KNN is classified as an instance-based learning algorithm, signifying its reliance on storing the entire training dataset for reference. When presented with a new data point for prediction, KNN calculates distances between this point and all data points in the training set, typically using a chosen distance metric (e.g., Euclidean distance). The algorithm then identifies the *k* nearest neighbors based on these distances. For classification tasks, KNN assigns a class label that is most prevalent among these neighbors. In regression tasks, it calculates the weighted average of the target values of the *k* neighbors. The choice of the distance metric is crucial and depends on the problem of interest. While Euclidean distance is widely used, alternatives like Manhattan distance or Cosine similarity can be employed to better suit specific data characteristics. KNN provides a versatile modeling approach that can effectively capture complex decision boundaries. The ability to handle intricate relationships makes it a potential candidate for applications in organic waste treatment, where the interactions among various factors can be complex. However, it’s essential to consider the specific characteristics of the organic waste treatment data. Additionally, while KNN excels in flexibility, it may face challenges when dealing with high-dimensional data and large datasets due to computational costs. Considering the potential richness and complexity of data in organic waste treatment scenarios, it becomes crucial to assess whether these challenges align with the nature of the dataset.

## State-of-the-art applications

### Data-driven modeling and optimization for thermochemical technologies

The usage of ML methods for thermochemical techniques e.g., gasification^16^, pyrolysis^16^, hydrothermal treatment methods^55^ have recently been reviewed by several researchers. These ML models developed were combined with optimization algorithms (e.g., genetic algorithm (GA), particle swarm optimization (PSO), etc.) to solve goal-oriented optimization problem for various process variables. A subgroup of work enhanced the interpretability of the ML-based organic waste treatment models by using feature importance and partial dependence analysis to gain a deeper understanding of the process dynamics of key parameters.

For example, a prior work^64^ predicted the higher heating value of organic waste gasification process based on proximate and ultimate analyses using three different types of ML methods such as FNN, SVM, and RF, where RF model showed the best predictive performance against the testing dataset (coefficient of determination (R^2^) > 0.92). The superiority of the RF model compared to the other models was attributed to the ensemble learning process offered by RF via the bootstrap sampling method (see Section “Ensembled decision trees”), which reduced the effect of noisy feedstock composition data on model overfitting. Nevertheless, the work did not include of reactor types and operating conditions, which are essential predictor variables to generalize gasification models. Another type of research work^68,69^ developed an FNN to predict the chemical exergy (i.e., maximum extractable energy while interacting with surroundings) of syngas production from organic waste using ultimate composition of organic waste, gasification temperature, and mass basis ratio of steam to organic waste. Two of gasifier such as downdraft gasifier and bubbling fluidized bed gasifier were explored in these efforts. Several works^70–74^ has used ML methods to directly predict hydrogen yield from organic waste gasification process to promote waste-to-energy conversion. Hydrogen yield was predicted for supercritical gasification, chemical looping gasification, and conventional gasification based on organic waste composition, reaction parameters such as temperature, pressure, residence time, particle size, etc. The ML models explored in these works included NN, SVM, RF, ensembled tree, and GPR, KNN, where RF and ensembled tree algorithms deployed multiple parallel trees to reduce model overfitting, leading to an R^2^ > 0.97. In addition to the composition of syngas or hydrogen, prediction of the char and tar yields during gasification process is essential to understanding the interplay of solid, liquid, and gaseous product yields. This is also useful for combining ML-based gasification models into process optimization algorithms and *what-if* scenario simulators (e.g., Monte Carlo simulation). In this realm, several studies have simultaneously predicted char and tar yields in addition to the elemental composition of syngas for organic waste gasification^58,75–78^, although their predictive performance was somewhat inferior than the models developed for syngas yield models. This was attributed to the smaller sizes of the datasets for char and tar yield models, which warrants further developmental efforts. Some of the advanced studies^58,77^ has considered a wide pool of input parameters to capture the intricate relationships in the input-output feature space such as proximate, ultimate, and lignocellulosic composition, temperature, operation modes (continuous and batch), gasifying agent (air, steam, oxygen), reactor type, reactor bed material, usage of catalyst, and scale of operation (lab or pilot). Organic gasification processes with other integrated systems such as solar energy-driven gasifier^79^, combined cooling, heating, and power (CHP) production systems^80^ has also witnessed the use of ML methods for process optimization and intensification. For example, a prior work^79^ has used RF algorithm to simulate the key variables of solar-driven organic waste gasification process with high accuracy (R^2^ > 0.98). In another effort^80^, an organic waste gasification cycle was combined with heat pump and absorption chiller for combined cooling, heating, and power production. The trade-off between the exergy efficiency and NH_3_ production rate was revealed through an SVM-informed GA, signifying the applicability of synergistic interactions between ML-based process models and heuristic optimization methods for integrated gasification systems.

In contrast to the gasification process where syngas is the primary product of interest, pyrolysis enables balancing the trade-off between the solid (biochar), liquid (bio-oil), and gaseous (pyrolytic gas) output products. Based on the usage, different variants of pyrolysis processes are adopted in the industry that promote yield of a certain type of product. Being a complex, multi-step thermochemical process, intricate kinetic modeling of pyrolysis becomes a non-trivial task, where researchers have deployed data-driven ML algorithms in recent years. In most of the cases prediction of biochar and bio-oil yields and compositions based on the input parameters covering ultimate, proximate, and lignocellulosic composition of feedstock, pyrolysis temperature, heating rate, residence time, inert gas flowrate, and particle size of feedstock has been the central theme of ML model development^81–90^. While most of these works focused on mono-pyrolysis of a wide variety of feedstocks (e.g., crop residues, woody waste, sludge), several efforts have been put into development of ML models for co-pyrolysis. For example, co-pyrolysis of organic wastes with polymeric waste^91^, biomass pyrolysis coke with rapeseed cake^92^, organic wastes with plastic waste^93,94^, and coal with organic waste^23,95^ have been studied. For these studies, an additional input parameter of interest considered in the ML models is the organic waste to co-pyrolytic material blending ratio, which significant affect the yield and compositions of the three-phase output products. Although slow and fast pyrolysis have been frequent choices for industrial deployments, MAP has attracted attention in recent years, for which development of ML models have been utmost essential. For example, a prior work^96^ has explored the utility of polynomial regression to model three-phase product yield during potassium hydroxide catalysed MAP of sawdust, achieving an R^2^ > 0.93. The model also captured the effect of feedstock pre-treatment using dry torrefaction. Despite the high R^2^ value, the work suffered from limited generalizability and robustness since it was built upon one specific type of feedstock with pre-determined feedstock treatment criterion. In contrast the XGBOOST model developed in ref. 97 had relatively a higher pool of feedstock information and achieved R^2^ > 0.9 for predicting both biochar yield and higher heating value (HHV) during MAP process. The high accuracy of the XGBOOST algorithm may be attributed to its pruning abilities and ensembled boosting to convert weak learners into strong learners, ultimately enhancing the performance on both training and testing datasets (see Section “Ensembled decision trees”). The study also included model-agnostic explainability analysis (e.g., using feature importance and partial dependence assessment), revealing that microwave power is one of the most important factors regulating biochar yield and HHV. A follow-up work^98^ on ML modeling of MAP utilized 249 datasets from the literature to explore three different types of models SVM, RF, and gradient boosting machine (GBM), where GBM achieved an R^2^ > 0.83. Although the GBM had working principles like the XGBOOST algorithm and leads to somewhat similar predictive performance, the training time of GBM was significantly longer than XGBOOST. This work also included extensive feature importance analysis, which indicated that temperature, microwave power, and reaction time were the key parameters. Although majority of the ML modeling works predicted the yield and composition of the biorefinery products (e.g., biochar, bio-oil, and pyrolytic gas) during different types of pyrolysis, a few has deployed ML methods for deciphering the kinetic mechanisms and parameters of pyrolysis^5,92,99–102^. For example, an NN-based model^99^ was developed to predict the unknown kinetic parameters (activation energy (*E*_*a*_), frequency factor (*A*) and order of reaction (*n*)) specific to thermogravimetric experiments of different feedstocks relevant for organic waste pyrolysis. The input parameters for this predictive model were proximate, ultimate, and lignocellulosic composition, and heating rate (*β*). The model predicted kinetic parameters for limited range of feedstocks (sawdust, wood, Areca nut husk, and banana leaves) with R^2^ > 0.97. In other instances^100,102^, a relatively extensive datasets were developed to predict activation energy of a wide range of feedstocks using multi-linear regression (MLR), RF, FNN, and SVM, where RF was able to capture the complex trends in activation energy and led to superior performance. Due to multi-scale nature of pyrolysis, an essential challenge in process simulation via computational fluid dynamics (CFD) is the consideration of interparticle heat transfer and chemical reactions^5^, which exponentially increase the computational time of the simulation. This issue was addressed by coupling an FNN-based particle simulation model of pyrolysis kinetics with full-scale CFD simulations. The approach significantly reduced the computational time of CFD simulation and resulted in an averaged bio-oil prediction error of 6.4%. Another research effort developed an FNN-based correction model to account for external heat transfer, particle diameter, and pyrolysis reaction mechanism^101^. Various input parameters for the data-driven correction model were heat transfer coefficient, particle size, gas temperature, moisture content, and dimensionless temperature of the particle. Overall, the data-driven parameter correction model facilitated zero-dimensional model-coupled lumped kinetic simulations for pyrolysis processes. This made it possible to embed key physical phenomena in ML models, enhancing its extrapolation capabilities.

The hydrothermal treatment technologies include a variety of treatment routes based on the desired output product (e.g., HTG, HTL, and HTC), thus requiring significant efforts towards unified ML model development^55^. Based on the types of hydrothermal treatment strategy the dominant output product mix varies across hydrochar, bio-oil, or hydrothermal syngas. The fraction of product for each choice of technology is regulated by a suite of input parameters which include organic waste compositions (elemental, proximate, biochemical, and ash), operating conditions (temperature, pressure, residence time, solid content, heating rate, catalyst, and inert gas type), solvent selection (reaction and product extraction solvents), and reaction mode (continuous and batch)^103^. Based on these parameters and ML model for hydrothermal treatment strategy predict the yield, elemental compositions, material characteristics, and fuel property of the output three-phase product mix. ML models have been developed in recent years to predict HTC-derived hydrochar production^104–112^, HTL derived bio-oil production^113–121^, HTG-based syngas generation^66,122,123^, or aqueous phase prediction^124^. For example, HTG produced syngas yield and composition based on a wide range of feedstock have been predicted using popular ML approaches such as CNN, FNN, GBM, XGBOOST, and RF, where both RF and XGBOOST algorithm had R^2^ > 0.85^66^. Since the work explored a relatively large dataset (~250) with a wide spread of the predictor variables, the NN-based models suffered overfitting and had lower accuracy. This suggests that ensembled tree algorithms (e.g., RF and XGBOOST) are essential to tackle such complex parametric trend and overfitting issues simultaneously. A related work has coupled GBM with PSO algorithm for HTG based syngas production based on an ASPEN software-simulated dataset. The hybrid model achieved R^2^ > 0.9, while feature importance analysis revealed that gasification temperature strongly regulates the variations yield and compositions of syngas^122^. A customized optimization-oriented artificial intelligence-based tuneable decision support system (TDSS) for HTG process was developed using more than 500 datasets. An adaptive multivariate RF with adaptive weighted rank aggregation was used as the backbone of the TDSS for predicting output parameters of HTG process^123^. A range of ML models has been developed to predict the hydrochar production via mono-HTC (i.e., with one type of feedstock)^105^ or co-HTC (i.e., which blends two different feedstocks)^110,125^ from a variety of feedstocks. It is important to note that ML model development for mono-HTC for popular waste streams such as sewage sludge, lignocellulosic waste, municipal solid waste, woody waste, and food waste has been developed^104–112^. These works have frequently used model explainability analysis coupled (SHAP, permutation importance, partial dependence analysis) ML model selection pipelines, which led to high accuracy (R^2^ > 0.9) interpretable model development. In some recent efforts the power of deep learning (DL)^104^ and natural language processing (NLP)^126^ have been leveraged to develop unified models for hydrochar (i.e., HTC derived biochar) and pyrochar (i.e., pyrolysis-derived biochar) production. These efforts are true examples of unifying multiple organic waste treatment processes into a single ML model for developing rapid prediction tools. For HTL process-based bio-oil (or bio-crude) production optimal ML models selection tools have been developed that showed superior performances (i.e., R^2^ > 0.9) of sophisticated techniques such as RF^114,117,120^, GBM^115,116^, GPR^118^, and XGBOOST^119^. All the algorithms have capabilities of tackling overfitting issues under noisy datasets, while the GPR algorithm had additional incentive of providing the predictive uncertainty in bio-crude yield. While most of the studies predicted yields of syngas, bio-oil, or hydrochar produced via hydrothermal treatment methods, the models predicting wastewater properties (e.g., pH, total organic carbon, total nitrogen, and total phosphorus) are essential towards understanding the adverse effects of wastewater pollutants on hydrothermal treatment processes. This was addressed by XGBOOST modeling of hydrothermal treatment processes to predict the aqueous phase properties with such input information as elemental and biochemical compositions of feedstock and reactor operating conditions^124^.

### Data-driven modeling and optimization for biochemical technologies

In numerous instances, researchers have used ML methods to decipher the process dynamics of biochemical technologies such as AD^7,34^, dark fermentation^36,127^, and composting^52^. Compared to thermochemical processes, these biochemical waste treatment methods adopt much lower temperatures (35–70 °C), which are suitable for a wide range of bacterial kinetics. This makes biokinetic modeling of the biochemical waste treatment a non-trivial task. ML models developed for these waste treatment processes have either been utilized for what-if scenario simulation or deployed in tandem with optimization algorithms for maximizing desired product yield or to increase long-term stability.

AD being one of the most complicated biochemical waste treatment techniques requires a real-time simulation tool to predict biogas (or methane) production, effluent characteristics, or process stability^7,34^. ML models help to correlate the input and output parametric spaces of the AD process in dynamic scenarios. The waste treatment process relies on a wide variety of feedstock and reaction parameters such as total solids, volatile solids, organic loading rate, pH, retention time, temperature, oxidation-reduction potential, electrical conductivity, alkalinity, ammonium nitrogen, volatile fatty acid (VFA), type of reactor, number of stages, reactor volume, scale of implementation, and C-to-N ratio. The output parameters for these models have generally been biogas yield, cumulative methane production, effluent characteristics, total ammonium nitrogen accumulation, or VFA production. Most of the earlier work has used FNNs to predict biogas and methane yields based on a very limited database of organic waste^128,129^, which makes their applicability questionable for other feedstock and operating parameters. Therefore, for developing data-driven models compatible with extensive feedstock databases, a wide variety of ML must be explored. In addition, since data-driven models are of black box nature, they must be coupled with model explainability analysis methods (e.g., feature importance, partial dependence assessment) to substantiate the relationship between predicted and predictor variables. These needs have necessitated the development of ML model selection pipelines for simulating anaerobic co-digestion (ACOD) with a wide variety of feedstocks. The efforts also included the implementation of online feature importance analysis, enabling the selection of critical process variables in real time, a first of its kind implementation in bioprocess modeling. For example, a prior work^35^ compared RF, GLMNET, SVM, and KNN algorithms to predict the methane yield based on feedstock compositions and reactor temperature. The KNN algorithm achieved the best performance with R^2^ = 0.73 and the model explainability analysis included MeanDecreaseGini- and InNodePurity-based feature importance analysis. Although it was counter-intuitive that sophisticated ensembled tree methods (e.g., RF) performed inferiorly than KNN, this might be attributed to the smaller size of dataset (~20) used in the work. This claim is further strengthened by another work^67^, which used a relative large pool (>50) of data exploring GLMNET, RF, XGBOOST, FNN, KNN, and SVM algorithms. In this case, the RF model showed the best performance (R^2^ = 0.82) for predicting methane yield, bolstering the claim that tree-based models are desired to be used for the regression/classification applications with relatively larger datasets. In addition to conventional input parameters for ML-based AD models (e.g., feedstock compositions and operating conditions), the study also incorporated genomic data to improve model prediction capabilities. Feature importance analysis revealed that genomic abundance data had a higher degree of influence on methane yield than operation conditions and feedstock information. Such ML models with explainability analysis helps to identify the most significant taxa, e.g., the ones that dictate the biochemical process efficiency. Subsequently, a tree-based ML pipeline optimization tool was developed for rapid prediction and modeling of ACOD for a municipal wastewater treatment plant located in the USA based on an eight-year period data (~2800 entries)^130^. The study explored twelve different ML algorithms including DT, AdaBoost, XGBOOST, RF, ExtraTrees, GBM, SVM, and KNN, among which ExtraTrees showed the highest accuracy (R^2^ = 0.72) in predicting methane yield. Due to the randomized node splitting, the ExtraTree algorithm achieved better model generalizability than other methods such as RF. This also resulted in a shorter training time than the RF model by saving an additional evaluation stage i.e., the optimal node splitting criterion method. Model explainability analysis for this work included permutation feature importance and single parameter partial dependence analysis, revealing the top five influential parameters as (a) waste content with COD > 20000 mg/l, (b) dairy waste content, (c) content of fat, oil, and gas, (d) rendering waste, and (e) amount of poultry blood. These parameters were either linearly or exponentially correlated to the methane yield, as revealed by partial dependence analysis. Such deep knowledge of parametric functional dependence is essential for combining ML models with optimization problems since the existence of non-convexity in these parametric spaces might lead to globally non-optimal predicted variables (*e.g*., biogas yield, VFA production, etc.) Similar ML model selection pipelines were developed subsequently by other researchers for simulating AD to predict methane yield, relative abundance of gene, and VFA, where SVM, FNN, GBM coupled with SVM^65^, and XGBOOST have been popular choices^65,131–134^. ML methods have also been combined with optimization algorithms to maximize biogas (or methane) yield or minimize the accumulation of VFA within the anaerobic digester^135–137^. FNN and GA have been integrated to determine the trade-off chart between biogas production and COD of the effluent^138^. These ML-integrated optimization studies have greatly facilitated the design optimization of the anaerobic digesters.

Dark fermentation, being an important waste-to-biohydrogen conversion process has low-temperature biochemical dynamics, requiring intricate tools for process simulation and optimization. To address this ML methods have been utilized in recent years to simulate biohydrogen production via dark fermentative routes^36,127^. Most of the earlier efforts toward developing ML models for dark fermentation were limited to FNNs^57,139–141^ based on input parameters such as chemical oxygen demand, pH, dark fermentation time, VFA, inoculum and substrate types, substrate and glucose concentrations, temperature, etc, while the output parameter was primarily biohydrogen yield or production rate. A subsequent effort^142^ compared response surface methodology coupled with FNN and SVM for modeling dark fermentation, which proved the superiority of the SVM method (R^2^ = 0.98) for predicting biohydrogen yields. Another latest study^143^ explored five different ML models (GBM, RF, AdaBoost, SVM, FNN) for predicting hydrogen yield based on a wide range of feedstock (wastewater) parameters, reaction conditions, etc. The work revealed that all three tree-based ensemble learning methods i.e., GBM (R^2^ = 0.98), AdaBoost (R^2^ = 0.91), and RF (R^2^ = 0.97), were suitable for predicting biohydrogen production via dark fermentation. Nevertheless, further ML modeling efforts are warranted towards enhancing the accuracy, model generalizability, and parametric explainability.

The composting strategy significantly differs from both AD and dark fermentation since composting is a method to store waste products sustainably rather than extracting valuable products from them. Therefore, capturing the process behavior via ML methods requires different approaches. Some of the prevalent parameters that control the composting process are temperature, moisture content, pH, electrical conductivity, nitrogen content, carbon content, and C/N ratio. ML methods for composting applications have mostly been used for predictive modeling and optimization purposes rather than reactor control operations as in AD or dark fermentation processes. For example, a prior work^144^ predicted the volume of biogas produced by spent mushroom and wheat straw composting using FNN, LR, and fuzzy inference system, where FNN outperformed the other two models with R^2^ > 0.98. Other works have also revealed the interest of researchers in predicting the CO_2_ produced by the organic waste composting process^51,145^. In another instance^146^, chicken manure and penicillin composting process was modeled using LR and RF models, revealing the quantities of humic acid, fulvic acid, and humus. For this scenario, the RF model outperformed the LR model proving the superiority of the tree-based methods. The total carbon removal efficiency of chicken manure and bagasse has been predicted using the FNN model with R^2^ = 0.99 with knowledge of input parameters such as time, antibiotic type, and wt.% of bagasse^147^. Composting maturity has also been an interest of predictive variables for kitchen waste based on time, temperature, pH, electrical conductivity, total nitrogen, C/N ratio, ammonia nitrogen, organic matter, nitrate content, and seed germination index^148^. Five different types of ML models such as LR, KNN, DT, SVM, and RF were deployed where RF showed the best predictive performance (R^2^ = 0.85). Recently, researchers have used GA-coupled FNNs to model the co-composting process of sewage sludge and biomass fly ash with excellent predictive performance^149^. Despite most of the work in the ML-based composting literature focused on predicting process variables, a few have also focused on ML-based microbiome analysis associated with the composting process. Compositing being a low temperature, microbiome-mediate decomposition process requires intricate modeling of bacterial gene abundance, which has mostly been overlooked to date. This is addressed in a recent study^150^, which analysed vital microbiomes using interpretable ML methods (i.e., ML accompanied by feature importance assessment), revealing *Bacillus*, *Acinetobacter*, *Thermobacillus*, *Pseudomonas*, *Psychrobacter*, and *Thermobifida* as dominant microbiomes. Towards process integration and alternative low-carbon technology installation efforts, the influence of incorporating an electric heating system with the compositing process has been studied using ML methods^151^. The weight reduction rate of the compost and energy consumption rate per kg of compost were the primary variables of interest. Two different ML models were explored including least squares and RF, where the least squares model outperformed (R^2^ = 0.89) the RF model. Due to the smaller size of the dataset, the RF model was prone to overfitting and counter-intuitively resulted in poor performance.

### Bioprocess controllers integrated with ML models

Organic waste treatment process control involves different input parameters and output state variables for different technologies. Gaining precise control on the input and state variables helps in process intensification, improves overall system performance and process stability. Conventional bioreactor control algorithms deploy state-space estimation using Kalman filter, detect anomaly using principal component analysis (PCA), or perform predictive control based on recursive least square algorithm^152,153^. However, deploying these approaches often result in large time-series error propagation, ultimately leading to transient deviation in the dynamic system^154^.

ML-based modeling and control algorithms can circumvent these drawbacks due to their robust time-series tracking capabilities. ML-based control algorithms have been applied to predict process dynamics of organic waste treatment systems. For example, one-dimensional CNN (1D-CNN) was used to develop a transient control model for pyrolysis reactor^155^. The gas phase components of the reactor that were controlled were methane, ethylene, ethane, propylene, and propane. The 1D-CNN significantly outperformed the partial least square methods and reduced dynamic fluctuations around the control setpoints. In another instance, a nonlinear autoregressive exogeneous NN (NARX-NN)—an advanced time-series forecasting technique was used to develop a predictive model accounting for essential parameters of biomass gasification such as gas composition, higher heating value (HHV), equivalence ratio and gas temperature^153^. The data-driven model was ultimately used in tandem with a model predictive controller (MPC) to solve different types of goal-oriented control problems. Overall, the control strategy kept the process conditions around 5% deviation around the setpoint. A similar type of work utilized an RNN-informed MPC for controlling the generation of hydrogen-rich gas and biochar from biomass waste gasification^156^. A related work compared the efficacies of NARX-NN, state-space NN, and Hammerstein-Wiener network for developing MIMO and SISO control schemes of gasification process. The NARX-based model outperformed both the other approaches, proving its superiority in time-series forecasting^157^. Data-driven algorithms for *e.g*., FNN have also been used to simulate process dynamics of ethanol production from sugarcane fermentation. The predictive model controlled the concentrations of ethanol and substrate with R^2^ values 0.97 and 0.92, with an error less than 7%^158^.

Sophisticated ML-based time series models have been used as MPCs for organic waste treatment processes (e.g., AD). The models included RF, FNN, extreme learning machine (i.e., FNN with adaptive weight correction capability), and SVM. The primary control parameter for these studies was VFA since its high concentration can significantly inhibit the methanogenesis process that facilitates production of biogas^159–161^. These data-driven models were integrated with statistical control charts to detect and classify anomality in AD systems, which is relevant for long-term operation of AD. Different types of graph-based NNs (e.g., graph convolutional network (GCN), deep belief network, stacked autoencoder, spatiotemporal GCN, etc.) were combined with MPC to improve the accuracies of these VFA prediction models. Feature selection capability was incorporated with these models to reduce the number of input parameters which ultimately reduced overfitting and improved predictive accuracies by up to 6.4%^162–165^.

### PINN integration with ML-based bioprocess models

NNs are often criticized of being black-box models, with limited understanding of physical phenomena, a concern pertinent within the bioprocess modeling community. Although integrating NNs with model-agnostic explainability analysis methods (e.g., SHAP, permutation feature importance, and partial dependence analysis) can provide knowledge on the dependencies of output variables on predictor variables, it still fails to account for physicochemical laws in the model. Due to the exceptional capability of embedding physical constraints/laws (e.g., conservations of mass, momentum, energy) into data-driven algorithms, PINNs have recently been used to model bioprocessing. For example, PINN was deployed to simulate the production of *β*-carotene using a fermentation process within a laboratory-scale batch reactor^166^. This approach circumvented modeling the complex multi-step kinetic mechanism of the fermentation process and enabled representing the dynamics via an experimental data-guided reduced order set of differential equations. The PINN was able to quantify the hidden (or unknown) parameters of the reduced-order model. In another instance, PINN was used to model the organic waste gasification process while considering the monotonicity of several physical parameters (e.g., equivalence ratio, moisture content, and temperature), ultimately predicting the contents of N_2_, H_2_, CO, CO_2_, and CH_4_ in the output gas^63^. The model achieved R^2^ > 0.9 and supported with explainability analyses methods e.g., partial dependence plots, physical consistency degree. The PINN developed in this work significantly outperformed commonly used ML-based regression models such as RF, GBM, XGBOOST, SVM, and FNN, proving the superiority of PINN. PINN has also been utilized to simulate the behavior of a laboratory-scale pyrolysis reactor with poly methyl methacrylate (PMMA) as a model waste/biomass^167^. The constructed PINN deciphered the unknown reaction parameters with great accuracy and constructed a data-driven model for further simulation purposes. Despite these recent efforts of applying PINNs to model bioprocesses and to establish true coordination between highly specific kinetic models (e.g., ADM1, Gompertz, acidogenesis-methanogenesis model^6^) and general-purpose data-driven models, the field is still in its initial stages. There is yet no evidence of using PINN for AD, compositing process, or hydrothermal treatment methods, which are essential to be developed in the future. Moreover, further efforts are required to improve the PINN models developed for gasification, pyrolysis, and fermentation with improved chemical mechanisms, thermofluidic models, and data-driven models with parametric uncertainty quantification.

### Life cycle assessment informed by ML models

An emerging application of ML-based models in bioprocess engineering for organic waste treatment is its integration with life cycle assessment (LCA) frameworks. LCA is a standard protocol that has been widely applied to evaluate the environmental impacts of a process, technology, system, or service by considering components and sub-processes throughout its whole life cycle. As an essential phase of LCA, life cycle inventory analysis (LCIA) about data gathering has a high requirement on the quality of input data which can significantly affect the outcome of LCA. It is essential to develop high fidelity, robust, and rapidly computable data-driven models, in which ML-based unit process modeling can play a key role. The current practices for gathering LCI data are usually a very rigorous process where discrete data from a large variety of literature is fed into the unit process model^29^. Often, the input parameters are not compatible with each other which deviates the unit process from reality. In several instances, model simulations are performed to gather LCI inventory, which is a time-consuming and uncertainty-prone process^168^.

ML-based unit processes have been developed to streamline the LCIA for different organic waste treatment processes. For example, an RF-based data-driven model was developed to facilitate the evaluation of the techno-economic and environmental performance of pyrolysis of three different organic waste (crop residues, woody waste, and wastewater sludge)^83^. The RF model predicted biochar yields, carbon content, nitrogen content, and higher heating values, which were essential to estimating carbon sequestration, nitrogen content for soil amendments, and direct biochar usage for heating applications, respectively. These datasets informed associated LCA which ultimately predicted the global warming potential (GWP) abatement of the development. ML models have also been utilized for assessing the environmental impact of bio-oil production via HTL process^169^. Four different types of ML models such as RF, GBM, SVM, and linear regression were compared. The GBM algorithm predicted the bio-oil yield with the highest accuracy (R^2^ = 0.92 and RMSE = 0.05), which was further combined with a model explainer, suggesting that the feedstock lipid content had the highest influence on the bio-oil yield. The model explainability analysis was carried out based on feature importance analyses (SHAP and permutation feature importance) and partial dependence analysis. The ML-informed LCA results quantified the life cycle generation of SO_2_, CO_2_, and NO_x_ for each kg bio-oil production. Another research work^170^ developed a unified ML model combining databases of HTL, HTG, and HTC for bio-crude, syngas, and hydrochar prediction, which was subsequently combine with an LCA model to evaluate GWP and energy-return-on-investment (EROI) metrics. The LCA framework also considered scenarios which integrated hydrothermal technologies with CHP and carbon capture with storage technologies. The study highlighted trade-offs between GWP and EROI for a wide variety of feedstock properties. In another instance, five different types of ML models were developed to inform LCA that was applied to assess the environmental benefits of an anaerobic digester integrated with an air-source heat pump (ASHP)^32^. The GPR model showed superior performance in predicting the biogas yield and methane content produced by AD. A feature importance analysis suggested that HRT, pH, and reactor operation temperature were the top three important parameters influencing the model output. The LCA compared two scenarios: the AD was heated with heat supplied by ASHP vs. heat supplied by a natural gas-fired boiler. The latter had up to 36% more GWP than the former. In another work, FNN was utilized to simulate Black Soldier fly larvae-based composting of kitchen waste^171^. Influences of essential parameters such as composting time, aeration frequency, number of larvae, composting container surface area, and waste composition on compositing performance were investigated using the FNN model. The data-driven model further informed an LCA assessment framework, which quantified 12 different LCIA metrics including GWP, human toxicity, acidification potential, etc. The FNN-informed LCA framework achieved accuracies up to 95.6%, proving the superiority of the methodology.

## Opportunities and recommendations

Although there have been significant advancements in data-driven ML modeling in the field of organic waste treatment, it is safe to conclude that it is still in its early days. Scepticism exists across industrial practitioners and academicians regarding the correctness of quantities predicted by ML models, posing several challenges and opening future research opportunities.

- The selection of the best possible ML model is found to be strongly correlated with the size of the dataset utilized. For example, the datasets with fewer than ~30 entries might suffice to fit well with simpler models (e.g., NN, SVM, and KNN) and result in overfitting if fed into ensemble-based methods. For relatively large bioprocess databases with O(10^2^) – O(10^3^), the benefits of ensemble methods such as RF and XGBOOST can be availed. This further calls for systematic rule generation for bioprocess modeling towards dataset-oriented model selection, which will mitigate overfitting.
- Most of the instances where ML models have been deployed for modeling organic waste treatment are based on datasets generated by lab-scale equipment, requiring extrapolation for industrial operations. Thus, the prediction uncertainty may increase, and the control system with the ML models may undergo instability. For such cases, model uncertainty quantification and adaptive error correction algorithm design become essential. In this realm, the uncertainty quantification power of GPR algorithms can be utilized for the development of the models.
- Most of the existing data-driven modeling works do not include interpretability analysis, making the models purely of black-box nature. There were several interpretability analysis efforts popularizing over the past few years based on feature importance assessment, partial dependence analysis, etc, which improved the understanding of the validity of ML modeling and the usage efficiency of existing data. In the future, it will be intriguing to explore other model-agnostic explainability methods to decipher the black-box models. An essential research direction in this area will be to develop real-time feature importance analysis towards parameter forecasting in MPC design for bioreactors and bioprocesses. Computational efficiency of such algorithms must also be tested using hardware-in-loop (HIL) systems before industrial deployments.
- Despite the immense promising capability of PINN for bioprocess modeling, their usage in this field has been extremely sparse. This often led to ML model predictions that do not comply with the conversation of mass, energy, or momentum. To mitigate this issue, governing equations can be used in tandem with the training loop for the ML algorithms. A research direction in this realm would be to integrate real-time AD data with first principal multistep kinetic models (e.g., ADM1, Acidogenesis-Methanogenesis model, etc.) using physics-informed ML approaches. This would ensure that the ML-predicted process variables comply with governing equations/laws by construction.
- Since the modeling of bioprocesses for organic waste treatment is a multi-disciplinary problem there must be strong synergy between chemical and biological kinetics, thermofluids, and genomics. However, many data-driven models developed omit at least one of the aspects, leading to oversimplification or biased consideration of essential process characteristics. Integrating information from all these domains would be required to construct a truly holistic data-driven model, which will be dependent on the types of organic waste treatment technologies as well as the application of the model developed.

## Conclusions

The diverse set of data-driven modeling techniques discussed in this study offers a comprehensive toolkit for a wide range of organic waste treatment technologies, which will ultimately contribute to achieving a net-zero pledge. The data-driven methods included sophisticated ML approaches such as NN, PINN, SVM, DT, RF, XGBOOST, KNN, and GPR, which have been widely used to model, optimize, control, and understand high- and low-temperature organic waste treatment methods. The usage of model explainability analysis methods which decipher the black-box contents of data-driven methods are discussed. The usage of ML methods in tandem with physical governing laws of waste treatment technologies enabled by PINN has shown to be a promising way to enhance model reliability. The need for deploying natural language processing-based ML models into the context of organic waste treatment strategies was also highlighted. An extensive search of the literature suggested that synergistic interactions of ML models with control algorithms, heuristic optimization engines, and life cycle assessment framework will aid holistic model development and promote greater resource circularity for organic waste management principles.

## Data Availability

No datasets were generated or analysed during the current study.
